# Toward the Use of Genomics to Study Microevolutionary Change in Bacteria

**DOI:** 10.1371/journal.pgen.1000627

**Published:** 2009-10-26

**Authors:** Daniel Falush

**Affiliations:** Department of Microbiology, University College Cork, Environmental Research Institute, Lee Road, Cork, Ireland; University of Toronto, Canada

## Abstract

Bacteria evolve rapidly in response to the environment they encounter. Some environmental changes are experienced numerous times by bacteria from the same population, providing an opportunity to dissect the genetic basis of adaptive evolution. Here I discuss two examples in which the patterns of rapid change provide insight into medically important bacterial phenotypes, namely immune escape by *Neisseria meningitidis* and host specificity of *Campylobacter jejuni*. Genomic analysis of populations of bacteria from these species holds great promise but requires appropriate concepts and statistical tools.

Bacteria lack a natural reproductive system, comparable to meiosis in eukaryotes, that segregates genes randomly. Instead, they evolve progressively through mostly small genetic changes, a proportion of which have noteworthy phenotypic effects. Some phenotypes are intrinsically difficult to study in the laboratory: virulence in humans or adaptation to particular ecological niches, for example. For these traits in particular, a promising avenue for scientific investigation is to identify the genetic changes that have provided the basis for their evolution in natural populations.

Most human phenotypes are hard to study in vitro and, consequently, methods for relating differences amongst humans to natural genetic variation are well developed. Association studies were proposed as an effective way of identifying genes with small phenotypic effects more than a decade ago [1] and, although initially controversial [2], the recent development of arrays for genotyping hundreds of thousands of single nucleotide polymorphisms (SNPs) scattered across the whole genome has allowed the approach to be successfully applied to many different human diseases and other phenotypes [3]. This success should inspire the development of equivalent protocols within bacteriology.

One challenge in developing generally applicable protocols for mapping phenotypic traits in bacteria is that processes by which microevolution occurs vary tremendously between species. For example, the human pathogen *Mycobacterium tuberculosis*, the causal agent of tuberculosis (TB), diverged recently from an obscure organism occasionally isolated from humans in Africa called *Mycobacterium canetti* [4]. *M. tuberculosis* shows very little variation and there is no evidence of strains acquiring DNA by import from other *M. tuberculosis* strains or indeed from any other organism, so that individuals are clones of each other, distinguished only by rare mutations or other small changes. By contrast, individual *Helicobacter pylori*, a cause of gastric cancer, acquire DNA from other members of the species at an extremely high rate. Consequently, as well as varying in gene content [5], strains isolated from different host individuals in the same ethnic group typically differ from each other at approximately 3% of nucleotides in core genes, and this diversity segregates nearly randomly [6]. The majority of bacterial species fall between these extremes, with their genomes showing signs of both clonal descent and DNA import from other strains.

In this essay, I will argue that the clonal mode of reproduction shared by all bacteria and Archaea, in which replication occurs by binary fission, in fact provides an extremely powerful context for association studies. These studies will require both appropriate technologies for genotyping and evolutionary analysis and judiciously chosen strain collections. I will here concentrate on two examples in which placing evolutionary changes in their clonal context provides the power to relate phenotype to genotype. Population-scale genome sequencing promises to allow a full and unbiased catalogue of variation within the same clonal context. This reconstruction will facilitate identification of loci that show correlations with phenotype or anomalous patterns that indicate natural selection, with minimal assumptions about the mechanisms by which phenotypes change.

## Example 1: Immune Escape during Clonal Spread of *Neisseria meningitidis*

*Neisseria meningitidis* lives in the human nasopharynx and is best known for its role in meningitis and other forms of meningococcal disease. *N. meningitidis* is a major cause of morbidity and mortality in childhood in industrialised countries and is responsible for epidemics, principally in Africa and Asia. Many lineages persist stably within human populations, causing little disease. There are a handful of “hyperinvasive” lineages, however, that have a distinct epidemiology, spreading rapidly from location to location and causing clusters of disease cases but not persisting in any one place.

Mark Achtman and colleagues examined variation within a single hyperinvasive lineage of *N. meningitidis*, designated subgroup III, over a period of three decades [7]. The strains within subgroup III showed little diversity in most of their housekeeping and other genes surveyed. A few loci were identified that did show variation, however, allowing clonal relationships to be partially reconstructed. This reconstruction demonstrated that there were strong bottlenecks during geographical spread, with a single ancestor for each major wave of infection. It also showed that, notwithstanding the low overall level of variation, certain genes encoding specific antigens changed repeatedly in different countries and pandemic waves.

**Figure 1:**
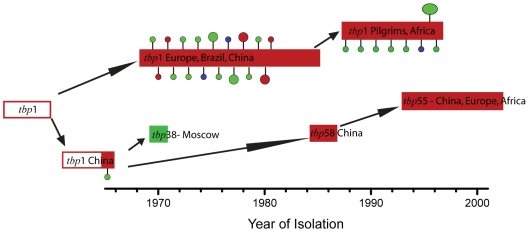
Acquisition of new *tbpB* genes by subgroup III *Neisseria meningitidis* during epidemic spread. Colours indicate the family of each *tbpB* allele, with red corresponding to family 4, green corresponding to family 1, and blue corresponding to family 3. The bars highlight the time frame, most common *tbpB* type, and geographical extent of each epidemic (in 1987, pilgrims from the Hajj pilgrimage briefly distributed the lineage worldwide). The circles correspond to variant genotypes. Small circles indicating that the variant allele was found in only one strain; large circles indicate it was found in between two and four strains.

The most remarkable variation was found in the transferrin-binding protein B gene (*tbpB*), which encodes a protein responsible for iron uptake that is expressed on the surface of the bacterium. This gene had evolved on three occasions by nonsynonymous point mutations that altered the structure of the protein and on 21 occasions by import of different versions of the protein from a variety of sources, including from *N. lactamica*, a closely related and entirely noninvasive species that also colonizes humans (Figure 1). The import events vary: analysis of similar *tbpB* changes in a closely related lineage showed that between 2 kb and 10 kb of sequence was transferred, which often altered the sequence of the flanking genes as well as *tbpB* [8]. In each case, however, an effect of the imported DNA was to change the externally exposed part of the protein from the usual version (called the family 4 version) to one of two antigenically highly distinct versions (family 1 and family 3).

The fact that functionally equivalent changes to *tbpB* are achieved by heterogeneous genetic events shows that the large number of imports is not caused by a recombination mechanism that is specific to the locus. Instead it reflects the amplifying effect of natural selection within the large number of bacteria that circulate during epidemics. Imports happen at a low rate throughout the genome, but those that cause an antigenic change at the *tbpB* locus have a selective advantage, meaning that they are observed at a much higher rate than imports elsewhere in the genome.

High diversity at a particular antigen locus is usually explained by invoking a mechanism called negative frequency-dependent selection [9]. Hosts who have been exposed to a particular variant develop immune responses against this variant. Bacteria with antigenically distinct variants escape this response, giving them an advantage in colonizing that host. At the population level, this selection should lead to the persistence of multiple variants. Yet, despite this selection for rare variants within individual epidemics, the antigenic diversity of subgroup III did not increase progressively over time but was instead reset at the beginning of each new epidemic, which was started by a strain with a family 4 allele.

The continuous generation of subgroup III strains with family 1 and 3 *tbpB* alleles is better explained by a mechanism called source–sink dynamics [10]. The source consists of an environment within which transmission of the bacterium is self-sustaining. Sinks consist of environments that bacteria can colonize effectively (perhaps by undergoing genetic modification) but from which onward transmission is ineffective. Here, the sink environment consists of individuals with acquired immunity to subgroup III strains that carry family 4 alleles, while the source is the remainder of the human population. The fact that the variant genotypes capable of colonizing the sink do not spread geographically but instead are repeatedly regenerated locally suggests that that these strains have reduced overall transmission fitness in naïve hosts, which comprise the majority of individuals in populations where an epidemic has not occurred recently.

Two other examples of sink environments are the lungs of immunocompromised patients for *Pseudomonas aeruginosa*, and the human urinary tract for *Escherichia coli* [10]; as for the *N. meningitidis* example, specific genetic changes have been identified that adapt strains of these bacteria to those environments but at the expense of overall transmission fitness, with the result that infections occur generally sporadically.

## Example 2: Host Specificity in *Campylobacter jejuni*

*Campylobacter jejuni* is a gram-negative bacterium commonly found in animal feces. It is often associated with poultry and naturally colonises the GI tract of many bird species. *C. jejuni* is one of the most common causes of human gastroenteritis in the world. Infection caused by *Campylobacter* species can be severely debilitating but is rarely life-threatening. Human infection is sporadic and, although poorly prepared food is often thought to be implicated, it is generally difficult to track the source. There has therefore been a substantial effort to isolate bacteria from a wide variety of reservoirs and to genotype them using multilocus sequence typing (MLST), which involves obtaining the DNA sequence for each isolate at a standardized panel of genes (seven for *Campylobacter*) that are chosen because they have an essential function and are present in the vast majority of isolates in the species [11].

The *C. jejuni* strains acquired by chickens are distinct from those of the wild birds around them, even when the poultry are kept outdoors [12]. Within farm animals, certain lineages are found with very different frequencies in chickens and cattle, whereas several genotypes are found at high frequency in both (strains with the MLST type ST-21, for example) [13]. Strains from different farm animals are more similar to each other than they are to strains found, for example, in starlings (a native European bird that is also common in may other countries, including the US) [14].

The digestive system of chickens differs from that of cattle in multiple aspects, and their body temperature is several degrees higher than that of cattle. This raises the question of how some lineages are able to compete successfully in both hosts. Mechanisms facilitating rapid phenotypic adaptation include: (1) inbuilt regulatory mechanisms that allow individual bacteria to alter gene expression in response to new environments [15], (2) “contingency loci” that mutate rapidly, creating phenotypic variation amongst bacteria that are otherwise genetically identical [16], and (3) import of DNA from other strains that are already adapted to the current environment.

A first step toward understanding the evolution of host specificity is to establish whether it is possible to predict the host origin of strains based on their genome sequence. One approach to doing this uses phylogenetic relationships. For example, the program AdaptML (http://almlab.mit.edu/ALME/Software/Software.html) attempts to assign branches of the phylogenetic tree to preferred habitats based on where the strains on that branch were isolated [17]. For *C. jejuni*, habitat can, for example, be equated to host species. The observation of a group of phylogenetically related strains in a single host species might reflect the common ancestor of those strains acquiring the traits required to survive in that species.

Since *C. jejuni* recombines frequently, the genome composition of each strain is determined by the sources from which it has imported DNA, as well by which strains it is phylogenetically related to. For example, ST-21, together with its variants, is a lineage analogous to subgroup III of *N. meningitidis*. Like subgroup III, the lineage has imported DNA from other strains on numerous occasions during its spread, with the result that many isolates have variant genotypes that differ from ST-21 at one or two of the seven MLST fragments. By convention, these strains are grouped with ST-21 into the ST-21 clonal complex.

**Figure 2:**
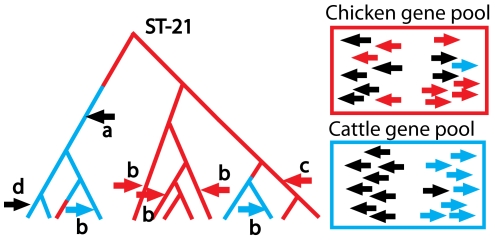
A schematic illustration of the evolution of the *C. jejuni* ST-21 clonal complex in cattle and chickens. The common ancestor of the complex occurred in chickens (red). During evolution, the lineage occasionally switched to a cattle host (indicated by a blue branch) and sometimes back to chicken. The bacteria acquired DNA by homologous recombination from other *C. jejuni* in the same host. Since recombination is assumed to occur from donors within the same host, the gene pool is determined by the genomic composition of the strains that colonize each host. The gene pools are illustrated for two separate loci (right and left facing arrows) in chickens and cattle. The gene pools contain alleles whose frequencies occur at much higher frequency in one host than another (shown in colour) and others that did not (shown in black). The former are informative about the host in which the recombination event occurred, while the latter are not. The recombination event labelled a introduces the left facing black arrow gene from the cattle gene pool and is phylogenetically informative because it defines a lineage that is largely restricted to cattle. The five recombination events labelled b are not phylogenetically informative, since they only affect a single strain in the sample. These events are nevertheless informative because they introduce alleles that are characteristic of the host species. The event labelled c is both phylogenetically informative and characteristic of host. The event labelled d is noninformative.

ST-21 itself has been found at high frequency in several agricultural species and elsewhere. Therefore, if a new strain is found to be ST-21, this provides little information on where it might have originated. However, for the variants of ST-21, Noel McCarthy and colleagues obtained significantly better than random assignment by predicting hosts based on the frequency with which the variant allele was found in chicken or cattle [13]. A useful signal of host-of-origin is thus provided by the DNA that each isolate has acquired (Figure 2). Furthermore, the high rate of recombination within particular hosts represents a mechanism by which complex adaptations to a particular host species can be acquired quickly subsequent to a host switch.

## The Power of Bacterial Genomics

Studies in bacteria have two major advantages over those in humans or other mammals when it comes to relating phenotype to genotype based on natural variation. The first is the magnifying effect of natural selection in enormous bacterial populations. This selection acts to rapidly increase the frequency of genotypes that give small fitness advantages in a particular environment, even if these genotypes are generated only rarely. Adaptation in bacteria is likely to be more frequent and to leave more distinctive genetic signatures than in species such as humans where signals of adaptation to local environments have proved to be remarkably subtle [18]. The second is the fact that evolution occurs in the context of progressively changing clonal backgrounds. This property can make it possible to identify strains that have extremely similar genomes but nevertheless differ phenotypically [19]. These strains represent the natural equivalent of an isogenic line and can allow precise inferences about the effects of natural variation and how different changes interact with each other.

In order to fully exploit the advantages of bacteria for detecting phenotypic associations, it is necessary to develop a conceptual and analytical framework within which rapid evolutionary change can be interpreted. One such framework is source–sink dynamics [10]. The *Neisseria* example illustrates the power of microevolutionary analysis in a source–sink ecological context to identify first the sink (hosts with immune responses to *tbpB* family 4 alleles) and second the loci under an immediate selective pressure to change within that sink (the *tbpB* gene).

Source–sink dynamics cannot be applied to investigate host specificity within *Campylobacter*, because individual host species, e.g., chicken, cattle, and individual species of wild birds, each harbour large, viable populations of bacteria with high rates of within-species transmission and do not represent sinks. Nevertheless, there is a key similarity between the *Neisseria* and *Campylobacter* examples, namely that the strains are repeatedly challenged by an environment that is novel in the recent history of the strain. In the *Neisseria* example, this challenge is repeatedly met by genetic changes at particular antigenic loci, which consequently have extremely atypical patterns of variation. In *Campylobacter* this challenge is met in the context of a high rate of import of DNA across the genome from other *Campylobacter* strains that already colonize the new host.

The availability of full genome sequences promises to enhance our understanding of the bacterial responses to new environments in a number of ways. First, phylogenetic relationships will be better resolved. In the *Neisseria* example, a well-resolved tree will elucidate patterns of transmission within epidemics and, for example, whether *tbpB* imports take place at the later stages of each wave and if strains with such imports ever reacquire family 4 alleles and seed later epidemics. In the *Campylobacter* example this will allow estimates of the number of occasions that the ST-21 lineage has jumped between host-species and establish whether there are sublineages that are becoming progressively more adapted to single-host transmission.

Second, genomics will provide a complete catalogue of loci whose pattern of descent is atypical of the genome as a whole and therefore either associated with a particular phenotype or putatively affected by selection. In the *Neisseria* example, an elevated rate of change at particular loci and consistency in the nature of those changes would provide signs of selection. In the *Campylobacter* example, loci that are imported at very high frequency and/or that are highly differentiated between host species may be involved in adaptation to a new host. An isolate-by-isolate analysis of the patterns of import should establish whether the multi-host lifestyle of ST-21 and, by extension, of *C. jejuni* as a whole is facilitated by import of DNA from locally adapted strains.

Third, genomics will allow detection of epistasis between loci. Epistasis occurs when the fitness effects of alleles at one gene are modified by the genotype at one or more additional genes. In outbreeding diploids, such as mammals, each allele has its fitness tested on a new genetic background in every generation, with the result that epistasis does not leave a distinctive signature in the frequency of particular combinations of alleles unless the loci are closely linked on the same chromosome or selection is very strong.

In bacteria, combinations of alleles remain together for many generations wherever they occur in the genome, providing ample opportunity for epistasis to bring particular combinations of alleles to high frequency. For example, subgroup III strains that have imported variant *tbpB* alleles can potentially enhance their fitness by importing other parts of the genome that adapt other strains in the *Neisseria* population to having high fitness when carrying family 1 or family 3 alleles. These parts of the genome could be detected by identifying parallel changes that have occurred on the 21 occasions that a variant *tbpB* allele was imported during the spread of subgroup III strains. Fitness interactions establish functional relationships between loci and represent a central part of the evolutionary landscape, for example triggering the origin of species [20]. Genome sequencing of bacteria should provide key insights on the nature of these interactions in natural populations.

In *C. jejuni* and other zoonoses, genomic analyses will facilitate a qualitative advance in our understanding of the epidemiology, ecology, and molecular biology of host switches. These developments will allow accurate delineation of the sources of human infection and an understanding of the factors promoting successful and pathogenic colonization of humans. In *N. meningitidis* and similar bacteria, we will gain a much better understanding of the genetic differences between invasive and noninvasive strains and the particular adaptive strategies that cause lineages to become invasive. These advances will together allow the design of targeted interventions that reduce the burden of human disease.

## Challenges for the Future

Advances in sequencing technology mean that it is becoming economically feasible to obtain complete or nearly complete genome sequences for large samples of bacteria. To better exploit this technology to understand bacterial phenotypes, the field should emulate the research program of human genetics and (1) develop statistical tools that use sequence variation to infer mechanisms of evolution [21] and patterns of genetic relationship [22]; (2) collect and sequence samples of isolates in which bacteria that differ in phenotypes of interest are matched as far as possible in time and space [23]; and (3) design statistical tools for detecting phenotypic associations [24] and natural selection [25] by identifying patterns of relationship at particular loci that are atypical of the genome as a whole.
